# A unified complex index to characterize two types of ENSO simultaneously

**DOI:** 10.1038/s41598-019-44617-1

**Published:** 2019-06-10

**Authors:** Zhiyuan Zhang, Baohua Ren, Jianqiu Zheng

**Affiliations:** School of Earth and Space Sciences, University of Science and Technology of China, Hefei, Anhui China

**Keywords:** Climate and Earth system modelling, Atmospheric dynamics

## Abstract

It is widely considered the El Nino-South Oscillation (ENSO) has several different types which can be simply classified as eastern Pacific (EP) type and central Pacific (CP) type. However, indices proposed so far can only characterize one single type of ENSO. In this paper, we develop a unified index which can characterize two types of ENSO simultaneously. The new index named as unified complex ENSO index (UCEI) is defined in the complex plane whose real part is NINO3 + NINO4 and imagine part is NINO3-NINO4. The modulus (r) and quadrants (θ) represent the ENSO strength and the ENSO types, respectively. Apart from the EP and CP types, the UCEI could further distinguish the MIX type of ENSO. Besides, the UCEI can capture the type-transforming processes within one ENSO event. Applying UCEI on historical events from 1950 to 2017 demonstrates the new index could be a very useful tool for the research of different types of ENSO.

## Introduction

El Nino-South Oscillation (ENSO) is the most influential pattern of the global climate with sea surface temperature anomalies (SSTA) over the tropical central and eastern Pacific Ocean. It is widely considered that the ENSO has several different types. Based on the SSTA distribution, it can be classified as the eastern Pacific (EP) type (or Cold Tongue type) and the central Pacific (CP) type (or Modoki type, Warm Pool type)^1–8^. The EP ENSO is the traditional type of ENSO which locates most of the anomalies over the eastern Pacific while the CP type known as the new type of ENSO which appears more frequently in recent decades has much more anomalies in the central Pacific^6^. For some events, the anomalies over the central and eastern Pacific are relatively both high which cannot be simply divided into EP or CP type and this type is generally called the MIX ENSO^7^.

The early indices, such as NINO4 (N4) [160°E-150°W, 5°S-5°N] and NINO3 (N3) [150°W-90°W, 5°S-5°N] are widely used to capture the anomalies in the central Pacific and eastern Pacific, respectively. However, N3 and N4 cannot well distinguish EP and CP ENSO as the anomalies in N3 (or N4) region will extend to the neighboring N4 (or N3) region. *Trenberth and Stepaniak* recognized the gradient between the central and eastern Pacific is necessary to completely describe ENSO and raised the Trans-Nino index (TNI) which is defined as the NINO12 subtracts N4^1^. Then *Ashok et al*. proposed the El Nino Modoki index (EMI) which uses the anomalies over the central Pacific subtract the eastern and western pacific^3^. *Li et al*. further adjusted the proportions of three regions of the EMI and proposed the improved El Nino Modoki index (IEMI) which could monitor the weak CP ENSO events better^9^. *Ren and Jin* developed N_CT_)/N_WP_ as a pair of indices to characterize EP and CP types of ENSO. The N_CT_ and N_WP_ are defined as N_CT_ = N3-αN4, N_WP_ = N4-αN3; α = 0.4 when N3*N4 > 0; α = 0 when N3*N4 < 0. The N_CT_ and N_WP_ demonstrate N3 and N4 could be suitable to distinguish EP and CP ENSO based on an appropriate combination^10^.

However, the existing indices can only characterize one type of ENSO. It is not convenient enough to determine the ENSO type as at least two indices are required. Not only that, in some cases, values of EP and CP indices are both high which makes the determination difficulty. So, whether one could develop a unified index that could characterize two types of ENSO simultaneously? In this paper, we introduce a novel index which could well achieve this purpose.

## Results

**Figure 1:**
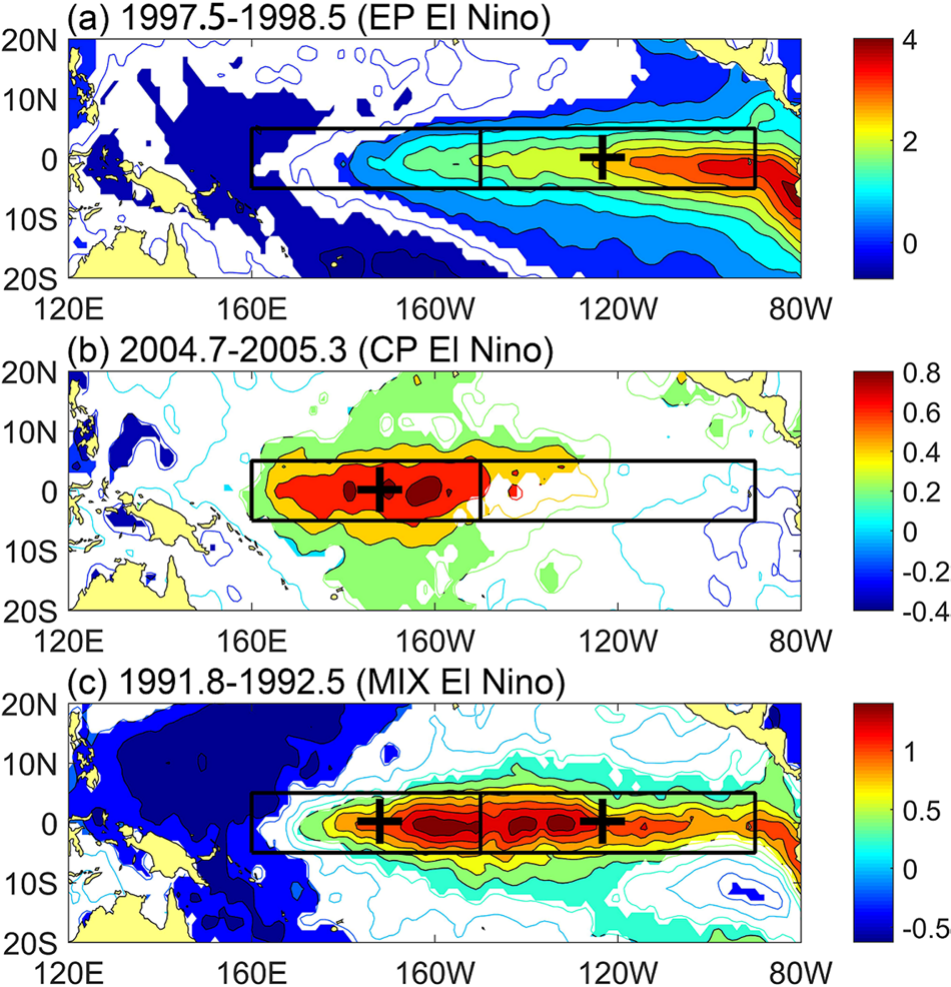
Examples of Averaged SSTA patterns of (**a**) EP El Nino, (**b**) CP El Nino and (**c**) MIX El Nino. Coloring areas passed 99% confidence level from a two-tailed Student’s t test. Two boxes indicate the regions N3 (left) and N4 (right), respectively.

To better descript how we construct the new index, we demonstrate the averaged SSTA distributions of 1997–1998, 2004–2005 and 1991–1992 El Nino events as the examples that represent the EP, CP and MIX types, respectively (Fig. 1). As the figure clearly shows that the warming anomalies of the EP El Nino mostly located in the region N3 and a small amount of anomalies extend to the region N4 (Fig. 1a). If we use N3 − N4, the result is definitely a positive number. In contrast, the warming anomalies of the CP type of El Nino mainly concentrate in the region N4 and very few anomalies appear in the region N3 (Fig. 1b). Then the result of N3 − N4 is a negative number. As for the MIX type of El Nino, the anomalies over the regions N3 and N4 are nearly equal (Fig. 1c). N3 − N4 is very small which could be regarded as approximately equal to 0. Taking into account the situation of La Nina, we further introduce the variable N3 + N4. The phase of N3 + N4 is positive for El Nino and negative for La Nina regardless of the type. Therefore, we could determine the type of El Nino (as well as La Nina) by the phase of N3 + N4 and N3 − N4 which can be summarized as follows:

1 $$\{\begin{array}{ccc}N3+N4 > 0 & N3-N4 > 0 & EP\,El\,Nino\\ N3+N4 > 0 & N3-N4 < 0 & CP\,El\,Nino\\ N3+N4 > 0 & N3-N4\approx 0 & MIX\,El\,Nino\\ N3+N4 < 0 & N3-N4 < 0 & EP\,La\,Nina\\ N3+N4 < 0 & N3-N4 > 0 & CP\,La\,Nina\\ N3+N4 < 0 & N3-N4\approx 0 & MIX\,La\,Nina\end{array}\}$$

**Figure 2:**
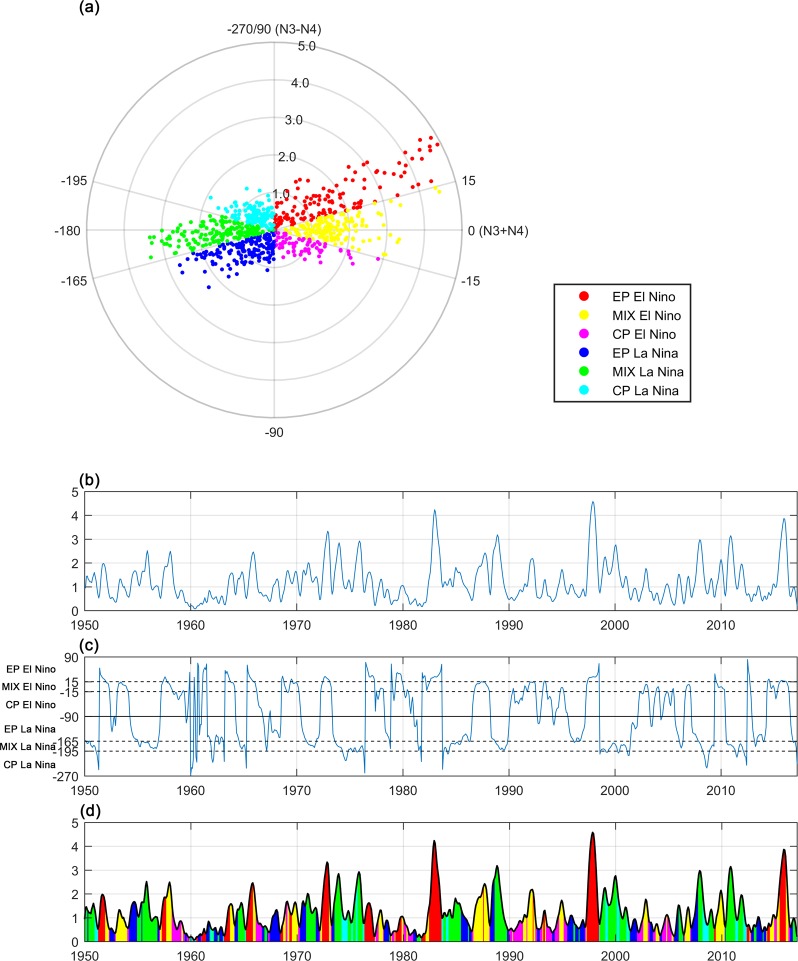
Scatter plots of UCEI (**a**), time series of r (**b**), θ (**c**) of UCEI and composite UCEI (**d**) from 1950 to 2017. ENSO types are denoted by different color. In this research, values of r that less than 0.5, between 0.5 and 1.0, 1.0 and 2.0, greater than 2.0 are determined as neutral, weak, moderate and strong, respectively.

On the basis of the above analysis, we introduce a complex plane whose real part is the N3 + N4 while imagine part is the N3 − N4. Figure 2a shows the monthly scatter plots of N3 + N4 and N3 − N4 in the complex plane from 1950 to 2017. As the Fig. 2a demonstrates the complex plane is divided into six regions that refer to EP, CP and MIX type of El Nino and La Nina respectively. In Practical application, the polar form is more suitable to descript ENSO events. Because in polar form, the strength of ENSO could be represented by r while the determination of ENSO types could be achieved by only θ. The the correspondence between θ and ENSO types is as follows:

2 $$\{\begin{array}{cc}\theta \in (15^\circ ,\,90^\circ ) & EP\,El\,Nino\\ \theta \in (\,-\,{\rm{15}}^\circ ,\,{\rm{15}}^\circ ) & MIX\,El\,Nino\\ \theta \in (\,-\,{\rm{90}}^\circ ,-\,{\rm{15}}^\circ ) & CP\,El\,Nino\\ \theta \in (\,-\,165^\circ ,-\,90^\circ ) & EP\,La\,Nina\\ \theta \in (\,-\,{\rm{195}}^\circ ,-\,{\rm{165}}^\circ ) & MIX\,La\,Nina\\ \theta \in (\,-\,{\rm{270}}^\circ ,-\,{\rm{195}}^\circ ) & CP\,La\,Nina\end{array}\}$$

It should be noted that the threshold of MIX type (15°) is not randomly selected. We assume the threshold of MIX type in the first quadrant is α. Larger the α, more significant the difference between N3 and N4 within the MIX region ((−α~α), (−180 − α ~ −180 + α)). According to this monotonic relationship, we change α from 1 to 90 and for each angle apply a two-tailed Students’s t-test on the N3 and N4 within the threshold. Statistical analyze demonstrates that when α < =15, difference between N3 and N4 is not significant while α > 15, difference is significant (at 95% confidence level). Therefore, 15 is a suitable threshold of MIX type of ENSO. We also examine the influence of asymmetry in El Nino-La Nina on the threshold of MIX ENSO, respectively. The threshold for MIX El Nino (La Nina) of all El Nino (La Nina) is 14° (17°), respectively.

After above analysis, we introduce a novel ENSO index called Unified Complex ENSO index (UCEI) which is defined as follows:

$$UCEI=(N3+N4)+(N3-N4)i=r{e}^{\theta i},$$

where

4 $$r=\sqrt{{(N3+N4)}^{2}+{(N3-N4)}^{2}}=\sqrt{2(N{3}^{2}+N{4}^{2})}$$

5 $$\theta =\{\begin{array}{cc}{\arctan }\frac{(N3-N4)}{(N3+N4)} & N3+N4 > 0\\ {\arctan }\frac{(N3-N4)}{(N3+N4)}-{\rm{180}} & N3+N4 < 0\end{array}\}$$

The r represents the ENSO strength while θ determines the ENSO type. Figure 2b–d display the time series of r, θ and UCEI from 1950 to 2017. A 3 month-running smoothing was applied for N3 and N4.

**Figure 3:**
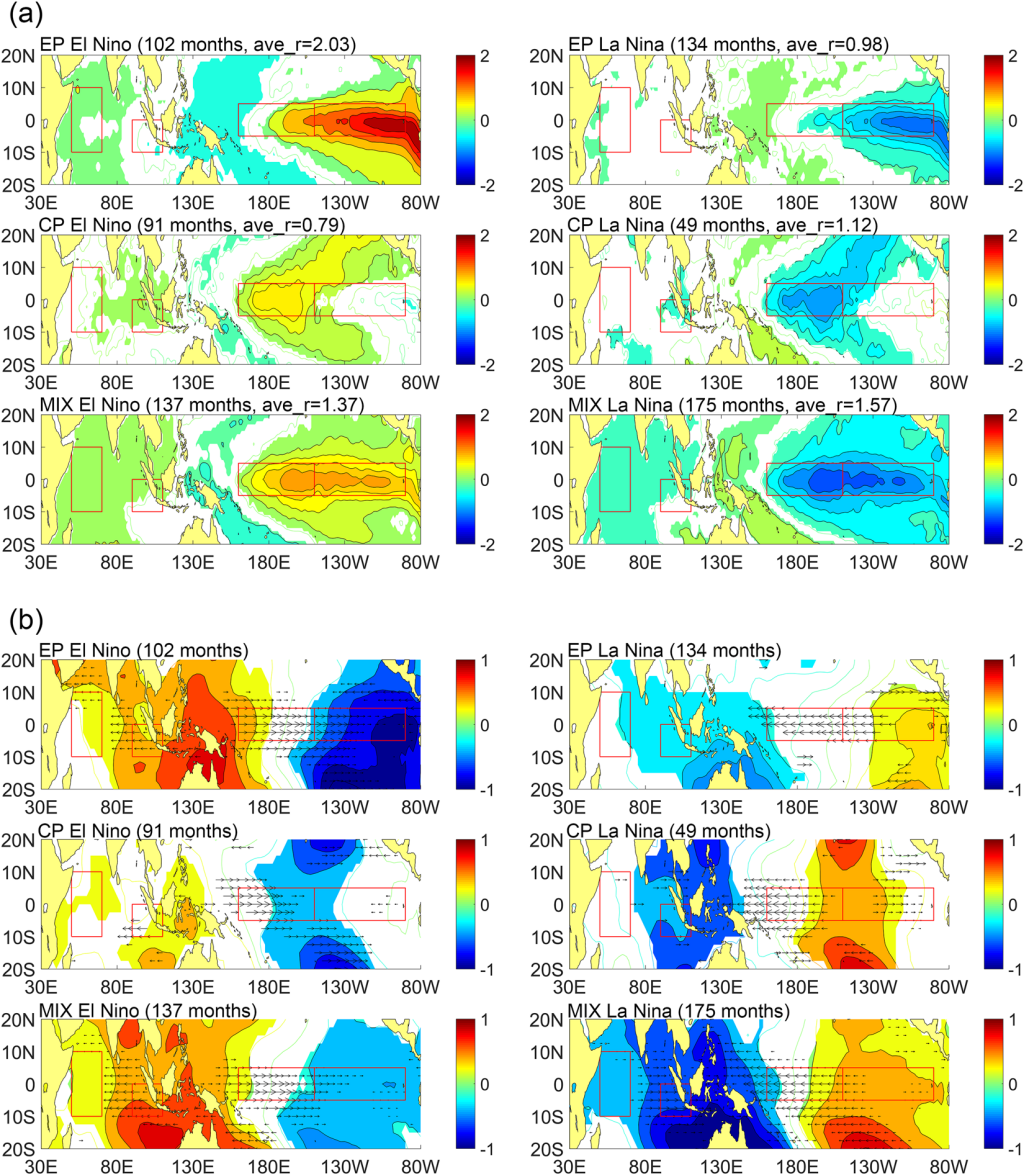
Composite SSTA (**a**), SLPA and UWNDA (**b**) distributions of different types of El Nino and La Nina classified by UCEI from 1950 to 2017. Coloring areas and vectors passed 99% confidence level from a two-tailed Student’s t test. Month numbers and averaged r of UCEI are shown in brackets. Red boxes indicate the regions IOD, N3 and N4, respectively.

Figure 3a shows the composite SSTA distributions of different types determined in Fig. 2a. Coloring areas passed 99% confidence level from a two-tailed Student’s t test. As we can see, even though the classification is based on only N3 and N4 regions, the composite distributions show the complete and typical EP, CP and MIX patterns in the whole tropical Pacific including the secondary feature regions such as the far eastern Pacific, off-equatorial regions and western Pacific which are consistent with the observation results. This demonstrates that the classification method of UCEI is very effective in distinguish different types of ENSO.

As Fig. 3a shows, the spatial distribution characteristics of El Nino and La Nina of each type are nearly the same. But Strength and frequency of occurrence are obviously asymmetry in El Nino and La Nina. Statistical results based on the UCEI demonstrate that the averaged strength of EP, CP and MIX El Nino are 2.03 (102 months), 0.79 (91 months), 1.37 (137 months). As for La Nina, the averaged strength for EP, CP and MIX types are 0.98 (134 months), 1.12 (49 months), 1.57 (175 months). Months that r less than 0.5 are determined as normal months and not selected. As we can see, the strength of EP La Nina is less than half of EP El Nino while CP and MIX types of La Nina are stronger than El Nino. In terms of frequency, CP La Nina appeared far less than CP El Nino while EP and MIX La Nina are more frequently than El Nino. All these lead to a result that EP and CP La Nina are both inactive compared with EP and CP El Nino and MIX type becomes the dominant pattern of La Nina. Previous researches also show that EP and CP La Nina are not clear as those of El Nino (Kug and Ham, 2011).

SSTA distributions in the Indian Ocean are also shown in the Figure. As the Fig. 3a shows, EP El Nino is related with warmer SSTA over western Indian Ocean than the eastern regions which indicates a positive Indian Ocean Dipole (IOD) while EP La Nina has few correlations to the Indian Ocean. In the case of CP El Nino, eastern Indian Ocean is warmer than the western regions which indicates a negative IOD. But the symmetrical correlation of CP La Nina in very weak. However, MIX El Nino and La Nina both have strong and broad correlation with the Indian Ocean that MIX El Nino/La Nina is linked with positive/negative IOD.

As the ENSO is a coupled ocean and atmosphere phenomenon, atmospheric features of different types of ENSO are also identified based on UCEI. Figure 3b is the same as 3a but for sea level pressure anomalies (slpa) and zonal wind anomalies (uwnda). EP El Nino has a strong dipole pattern with positive slpa widely spread over eastern Indian Ocean and western Pacific Ocean and negative slpa over eastern Pacific Ocean accompanied with easterlies over eastern Indian Ocean and westerlies over central Pacific Ocean. EP La Nina has a symmetrical but weaker pattern and no significant zonal wind anomalies over Indian Ocean. MIX El Nino has a similar pattern of EP El Nino but few gradients of negative slpa. The pattern of MIX La Nina is symmetrical to MIX El Nino. It should be noticed that MIX La Nina is the only type of La Nina that accompanied with westerlies over the Indian Ocean which are highly related to IOD events.

**Figure 4:**
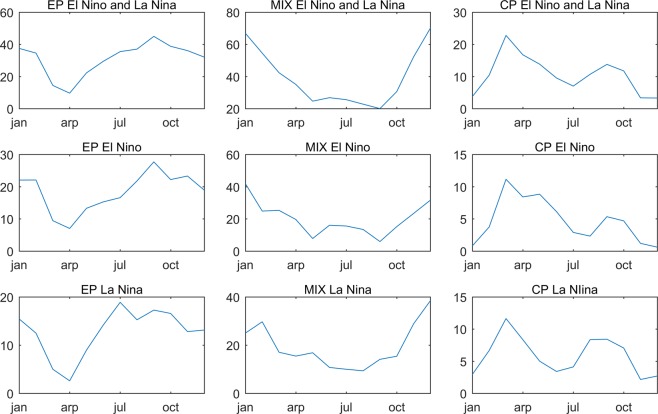
The sum of r based on calendar months of each type of El Nino/La Nina months from 1950 to 2017. Months that r < 0.5 are determined as normal months. ENSO type is determined by the θ of UCEI.

Phase locking properties of different types of ENSO are also identified by the UCEI. Figure 4 shows the sum of r based on calendar month of each type of El Nino/La Nina. As the Figure demonstrates EP ENSO is very weak in the spring while strong in the other seasons. MIX ENSO is strong in the winter while weak in the other seasons. CP ENSO is strong in the spring and autumn while weak in the winter and summer. As the Figure shows asymmetry in El Nino-La Nina is not obvious in the phase locking properties of ENSO.

**Figure 5:**
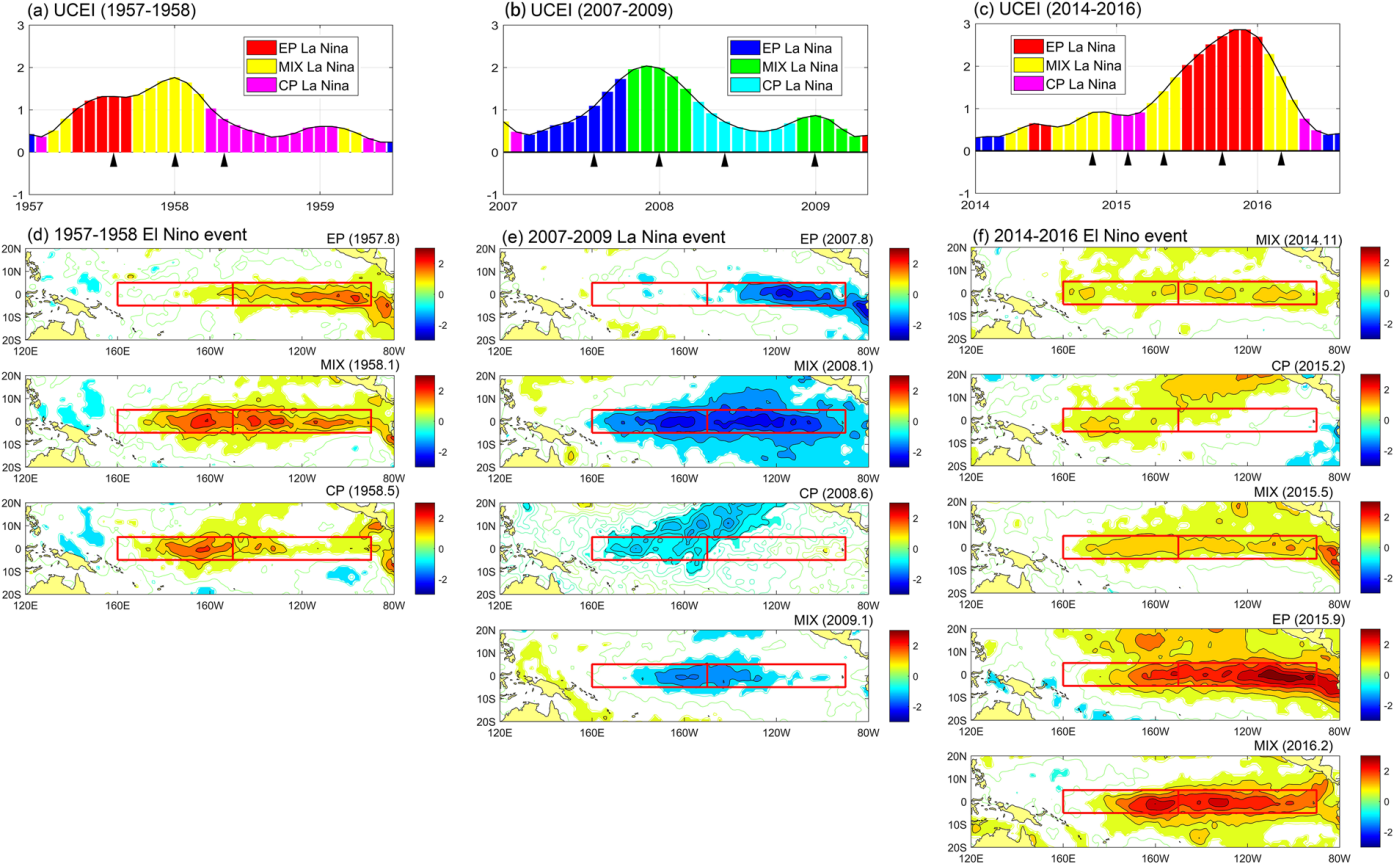
The UCEI evolutions during 1957–1958 El Nino (**a**), 2007–2009 La Nina (**b**) and 2007–2009 El Nino (**c**), respectively. Black arrows indicate time points of subplots in (**d–f**), respectively. (**d–f**) Are the SSTA distributions at different times corresponding to the (**a–c**) evetns. Values great than 0.5 and less than −0.5 are filled with color.

During ENSO events, the SSTA distribution is constantly changing with the evolution. And even in some cases, the ENSO type may be changed. A unique ability of the UCEI is that the new index could capture the type-transforming processes within ENSO event. As Fig. 5 demonstrates that some ENSO events have the type-transforming processes. For example, during 1957–1958 El Nino event (Fig. 5a,d), the development period pattern is EP type, then in the mature period transformed to MIX type, and finally in the decay period transformed to CP type. Duration of each period is long enough that the ENSO pattern cannot be described simply as EP (or CP) type. “EP->MIX->CP” is more suitable to describe the evolution of the SSTA pattern of this ENSO event. The 1965–1966 El Nino also experienced a similar “EP->MIX->CP” type-transforming process (not shown in Figure). The type-transforming also appeared in the La Nina event. For example, the 2007–2009 La Nina event (Fig. 5b,e), type transformed from “EP->MIX->CP->MIX”. The recent strong 2014–2016 El Nino event (Fig. 5c,f) also has an obvious type-transforming process which is “MIX->CP->MIX->EP->MIX”. The previous strong El Nino events are dominated by EP type but during 2014–2016 El Nino event, only mature period is EP type which implies the dynamical mechanisms and influence on global climate of this event may be very different from the traditional strong El Nino events. The UCEI can not only capture the type-transforming but also predict it. As the ENSO type is determined by the quantized θ, we could easily predict the trend of type-transforming in the next few months by the linear fit of θ. The historical ENSO events determined by UCEI from 1950 to 2017 are shown in Table 1. We examine the durations of three types of ENSO events that without type-transforming. The result shows the averaged durations of EP, CP and MIX ENSO are 14, 16 and 9 months which are close to the result of previous research that EP ENSO for 15 months and CP ENSO for 8 months^6^. We noticed CP ENSO only appeared 3 times during 1950–1977 but 9 times during 1977–2016 which demonstrates the tendency that CP type of ENSO appeared more frequently^3,6^.

**Table 1:** ENSO events determined by UCEI from 1950 to 2017.

No.	ENSO events	phase(strength)	Type
1	1950–1951	La Nina (Weak)	MIX
2	1951–1952	El Nino (Moderate)	EP->MIX
3	1953	El Nino (Weak)	MIX
4	1954–1956	La Nina (Moderate)	EP->MIX
5	1957–1959	El Nino (Moderate)	EP->MIX->CP
6	1963–1964	El Nino (Moderate)	EP->MIX
7	1964–1965	La Nina (Moderate)	EP->MIX
8	1965–1966	El Nino (Moderate)	EP->MIX->CP
9	1967–1968	La Nina (Weak)	EP
10	1968–1970	El Nino (Moderate)	MIX->EP->MIX
11	1970–1972	La Nina (Moderate)	EP->MIX->EP
12	1972–1973	El Nino (Strong)	EP
13	1973–1976	La Nina (Moderate)	EP->MIX->CP->MIX
14	1976–1977	El Nino (Moderate)	EP
15	1977–1978	El Nino (Weak)	CP->MIX
16	1979–1980	El Nino (Weak)	EP->CP
18	1982–1983	El Nino (Strong)	EP
19	1983–1986	La Nina (Moderate)	Mix->EP
20	1986–1988	El Nino (Moderate)	Mix
21	1988–1989	La Nina (Strong)	EP->Mix
22	1990–1992	El Nino (Moderate)	CP->Mix
24	1994–1995	El Nino (Moderate)	CP->Mix->CP
25	1995–1997	La Nina (Moderate)	EP
26	1997–1998	El Nino (Strong)	EP
27	1998–2001	La Nina (Moderate)	Mix
28	2002–2003	El Nino (Moderate)	CP->Mix
29	2004–2005	El Nino (Weak)	CP
30	2005–2006	La Nina (Moderate)	EP->Mix
31	2006–2007	El Nino (Weak)	Mix
32	2007–2009	La Nina (Moderate)	EP->Mix->CP->Mix
33	2009–2010	El Nino (Moderate)	Mix
34	2010–2012	La Nina (Strong)	Mix->CP->Mix
36	2014–2016	El Nino (Strong)	Mix->CP->Mix->EP->Mix

An El Nino or La Nina event is determined when the r of UCEI exceeds 0.5 for at least 5 months. The ENSO strength are categorized as weak, moderate and strong when the maximum of r belongs to (0.5, 1), (1, 2), (2, +∞), respectively. In order to eliminate short-term noise signals, ENSO type that lasts for no more than 3 months is ignored.

## Summary and Discussion

In this study, we develop a unified complex ENSO index (UCEI) which can characterize and distinguish EP and CP types of ENSO simultaneously. Based on the different features of EP and CP ENSO in regions N3 and N4, we construct the complex plane of N3 + N4 and N3 − N4. The El Nino type could be determined by the sign of N3 + N4 and N3 − N4. According to the significant test of difference between N3 and N4, the MIX ENSO could be further distinguished. Using the polar form, the ENSO type could be determined only by the argument (θ). And the ENSO strength could be represented by the modulus (r). Hence, we could characterize EP, CP and MIX ENSO simultaneously with the new index. As the previous indices can only characterize a specific type of ENSO, such an index will be a very convenience tool for the researches on the different types of ENSO.

Previous indices generally adopt the way that using strength of EP and CP type of ENSO to describe ENSO events. Among them, NCT and NWP^11^ have something in common with UCEI. They are both constructed by NINO3 and NINO4 without additional defined areas and complicated operations. The Correlation coefficients between r (r flips to –r when negative phase) and NCT, NWP, NCT + NWP are 0.89, 0.60, 0.99, respectively. As we can see, NCT or NWP only represents the decomposed strength of ENSO (EP or CP) which is not actual strength of ENSO. To determine the ENSO type, we also need a comparison of two indices. Therefore, the UCEI adopts a new way that using ENSO strength (not the component EP or CP strength) and ENSO type to describe ENSO events. The advantage of this way is that we can get the ENSO strength and types directly. Therefore, UCEI is more intuitive and convenient in practical application. However, the UCEI also has some deficiencies. For example, the generally used statistical analysis based on traditional indices such as auto-correlation, lead-lag correlation, and dominant frequency of each type of ENSO in the dynamical forecast models might be hard performed by UCEI.

## Data

The SSTA data used in this study is the monthly mean anomalies of Hadley Centre Sea Ice and Sea Surface Temperature data set^12^. We choose the time period from jan1950 to jan2017. The monthly mean zonal wind and sea level pressure data^13^ were from the NCEP reanalysis-derived data provided by the NOAA/OAR/ESRL PSD (Boulder, Colorado, USA) on their website at http://www.esrl.noaa.gov/psd/.
